# A Mouse Model to Study the Pathogenesis of γ-herpesviral Infections in Germinal Center B Cells

**DOI:** 10.3390/cells12242780

**Published:** 2023-12-06

**Authors:** Ursula Rambold, Stefanie Sperling, Zakir Chew, Yan Wang, Beatrix Steer, Krisztina Zeller, Lothar J. Strobl, Ursula Zimber-Strobl, Heiko Adler

**Affiliations:** 1 Institute of Asthma and Allergy Prevention, Helmholtz Center Munich, German Research Center for Environmental Health, Member of the German Center of Lung Research (DZL), 85764 Neuherberg, Germany; ursula.rambold@helmholtz-munich.de (U.R.); beatrix.steer@helmholtz-munich.de (B.S.); 2 Research Unit Gene Vectors, Research Group B Cell Development and Activation, Helmholtz Center Munich, German Research Center for Environmental Health GmbH, 81377 Munich, Germanyyan.wang@helmholtz-munich.de (Y.W.); krisztina.zeller@helmholtz-munich.de (K.Z.); lothar.strobl@helmholtz-munich.de (L.J.S.); 3 Institute of Lung Health and Immunity, Helmholtz Center Munich, German Research Center for Environmental Health, Member of the German Center of Lung Research (DZL), 85764 Neuherberg, Germany; 4 Walther-Straub-Institute of Pharmacology and Toxicology, Ludwig-Maximilians-University Munich, Member of the German Center of Lung Research (DZL), 80336 Munich, Germany

**Keywords:** MHV-68, conditional transgenic mice, GC B cells, PrimeFlow RNA assay, LMP2A, CD30

## Abstract

CD30-positive germinal center (GC)-derived B cell lymphomas are frequently linked to Epstein–Barr Virus (EBV) infection. However, a suitable animal model for the investigation of the interplay between γ-herpesvirus and host cells in B cell pathogenesis is currently lacking. Here, we present a novel in vivo model enabling the analysis of genetically modified viruses in combination with genetically modified GC B cells. As a murine γ-herpesvirus, we used MHV-68 closely mirroring the biology of EBV. Our key finding was that Cre-mediated recombination can be successfully induced by an MHV-68 infection in GC B cells from Cγ1-Cre mice allowing for deletion or activation of loxP-flanked cellular genes. The implementation of PrimeFlow RNA assay for MHV-68 demonstrated the enrichment of MHV-68 in GC and isotype-switched B cells. As illustrations of virus and cellular modifications, we inserted the EBV gene LMP2A into the MHV-68 genome and induced constitutively active CD30-signaling in GC B cells through MHV-68 infections, respectively. While the LMP2A-expressing MHV-68 behaved similarly to wildtype MHV-68, virally induced constitutively active CD30-signaling in GC B cells led to the expansion of a pre-plasmablastic population. The findings underscore the potential of our novel tools to address crucial questions about the interaction between herpesviral infections and deregulated cellular gene-expression in future studies.

## 1. Introduction

The γ-herpesviruses Epstein–Barr Virus (EBV) and Kaposi’s sarcoma-associated herpesvirus (KSHV) are implicated in human lymphomagenesis. Approximately 30% of Hodgkin lymphomas (HL) are EBV-positive, and nearly all Primary Effusion Lymphomas (PEL) are KSHV-positive with some cases being both KSHV- and EBV-positive [1,2,3]. Moreover, CD30^+^ Diffuse Large B cell Lymphomas (DLBCL) are significantly associated with EBV [4] and in endemic regions more than 90% of Burkitt’s lymphomas are associated with EBV infections [5].

The precise mechanisms by which γ-herpesviruses contribute to lymphoma development are not fully understood. To better understand the interactions between viral and host genes during lymphoma development, it would be invaluable to have a manipulable animal model that allows for the genetic modification of both the virus and host cells. To date, such studies have been impeded by the inability of EBV and KSHV to infect murine cells. A potential solution lies in the murine γ-herpesvirus-68 (MHV-68), which mirrors EBV in its viral strategies, including virus replication and establishment of lifelong persistence [6]. 

Shortly after infection, EBV establishes the so-called latency III program in which six nuclear viral proteins (EBNAs) and three membrane viral proteins (LMPs) are expressed, promoting unlimited B cell proliferation [7,8]. To evade the immune response, EBV downregulates these proteins, establishing a final latency in memory B cells (latency I/0). An intermediate latency (latency II) is observed in GC B cells, where the viral proteins LMP1, LMP2A and EBNA1 are still expressed. EBNA1 is essential for the replication of the viral episome [9,10]. LMP1 supports GC B cell survival by mimicking a constitutively active CD40 [11,12,13,14,15] while LMP2A mimics a B cell receptor (BCR) and is able to rescue GC B cells that lost their BCR during somatic hypermutation [16,17,18,19]. LMP1 and LMP2A are expressed in most EBV+ lymphomas implying that LMP1 and LMP2A contribute to lymphomagenesis through their pro-survival effects [20]. 

MHV-68, like EBV, traverses through GCs to establish its final latency in memory B cells [21,22]. Nevertheless, in contrast to EBV, MHV-68 lacks the expression of key EBV proteins such as LMP1 and LMP2A [6]. This distinction may explain why, in contrast to EBV, MHV-68 is not definitively associated with the development of B cell lymphomas in mice. Nevertheless, the genetic manipulability of MHV-68 offers the opportunity for the creation of recombinant viruses. Notably, the integration of EBV genes LMP1 and LMP2A into the MHV-68 genome can be accomplished through bacterial artificial chromosome (BAC)-based recombineering, allowing for the incorporation of EBV characteristics into the MHV-68 framework [23]. 

EBV infections trigger the dysregulation of cellular genes, potentially cooperating with viral genes in EBV pathogenesis. An example is the upregulation of CD30 expression, a member of the tumor-necrosis-factor-receptor (TNF-R) superfamily. The majority of EBV-associated lymphomas such as HL [2], PEL [3], and a subgroup of DLBCL [4], express high levels of CD30, whereas in normal lymphoid tissues, CD30 is expressed by only a few activated B lymphocytes found in the GC and the extrafollicular region, characterized by high proliferation and a c-myc expression profile [24]. Our recent findings provide evidence that aberrant CD30 expression promotes B cell expansion thereby contributing to lymphomagenesis [25]. 

In this study, we aimed to explore the utility of MHV-68 for investigating the collaboration between herpesviruses and deregulated cellular genes in EBV-pathogenesis. Given that most EBV-associated lymphomas originate from the germinal center (GC) or post-GC B cells [3,5,20,26], we focused on studying viral infections in combination with deregulated gene expression in GC B cells. To this end, we employed conditional transgenic mice along with the GC-specific Cγ1-cre strain, known for inducing Cre-mediated recombination in GC B cells upon Thymus-dependent (TD)-immunization [27]. The primary objective was to assess if MHV-68 infection triggers Cre-recombination in GC B cells. By using the PrimeFlow RNA method [28,29,30], which targets the t-RNA-like sequences of MHV-68, we then focused on characterizing the B cell populations, which contained the virus. We showed that an MHV-68 infection can trigger Cre-mediated recombination in combination with Cγ1-Cre mice. However, there seemed to be no direct correlation between cells mediating Cre-mediated recombination and cells containing the virus. As an illustrative example of probing the impact of γ-herpesviral infections on the pathogenesis of EBV-related diseases, we engineered a recombinant virus, MHV-68 LMP2A, and induced deregulated CD30 signaling in GC cells upon MHV-68 infection. Our study offers initial insights into the potential of this innovative approach for investigating the interplay between deregulated cellular gene expression and γ-herpesviral infections in the development of γ-herpesvirus-associated lymphomas in mice.

## 2. Material and Methods

### 2.1. Mice

C57BL/6J mice were purchased from Charles River Laboratories (Sulzfeld, Germany). R26/CAG-CARΔ1^StopF^ reporter mice, generously provided by the lab of Marc Schmidt-Supprian [31], were engineered by incorporating a truncated form of the human coxsackie/adenovirus receptor CAR into the *rosa26* locus. The CAR expression was controlled by the CAG promoter and a loxP-flanked stop cassette positioned between the promoter and the *car* transgene. To ensure CAR expression in GC B cells, R26/CAG-CARΔ1^StopF^ reporter mice were bred with the Cγ1-cre mouse strain [27] resulting in the generation of the R26/CAG-CARΔ1^StopF^//Cγ1-cre (CAR//Cγ1-cre) mouse strain. TD-immunization of these mice induced Cre expression mainly in GC B cells and subsequent removal of the stop cassette leading to the expression of the reporter CAR (Cre-reporter). Staining for CAR allowed for identification of cells which performed Cre-mediated recombination. LMP1/CD30^stopfl^ mice were created in our laboratory by inserting a transgene that fused the N-terminal and transmembrane domain of LMP1 with the signaling domain of CD30 [25]. The LMP1 transmembrane domain induced aggregation of LMP1 in the plasma membrane initiating ligand-independent constitutively active CD30 signaling. The *lmp1/cd30* transgene was regulated by the endogenous rosa26 promoter and a loxP-flanked stop cassette positioned upstream of the transgene. Similar to R26/CAG-CARΔ1^StopF^ mice, LMP1/CD30^stopfl^ mice were crossed to Cγ1cre mice, resulting in LMP1/CD30//Cγ1-cre (LC30//Cγ1-cre) mice. Immunization of these mice resulted in expression of Cre in GC B cells, removal of the stop cassette, and expression of the transgene *lmp1/cd30* along with the reporter gene *hcd2*, controlled by an IRES element located downstream of the *lmp1/cd30* transgene. This strategy ensured constitutive CD30 signaling in both germinal center (GC) and post-GC cells. Cells that had performed Cre-mediated recombination and expressed the transgene LMP1/CD30 could be traced by staining for hCD2. CAR//Cγ1-cre and LC30//Cγ1-cre mice were on a Balb/c background. The mice were bred and maintained in-house under specific pathogen-free conditions. Mice were 8–18 weeks old when used for experiments. The experiments were approved by the government of Upper Bavaria and performed in compliance with the German Animal Welfare Act. 

### 2.2. BAC Mutagenesis of MHV-68 hNGFR and MHV-68 LMP2A

SFFV-hNGFR was cut out of the pCDH-SFFV-hspCas9-T2A-NGFR plasmid (kindly provided by Prof. Dr. Irmela Jeremias, Helmholtz Center Munich) to insert the sequence into the MHV-68 genome. First, EcoRI and NsiI enzymes were used to remove hspCas9-T2A region from the plasmid. This was followed by a ClaI and SalI digest to excise SFFV-NGFR out of the plasmid. The sequence was then cloned into the PmlI site (nucleotide position 46,347 of the MHV-68 genome) of the plasmid pST76K-SR [23] containing a 4.0 kb SphI-SacI fragment of MHV-68 (nucleotides 44,301 to 48,346). As a result, SFFV-hNGFR was flanked on both sides by homologous sequences as needed for homologous recombination during the two-step BAC mutagenesis procedure. 

Similarly, a fragment containing LMP2A downstream of the HCMV promoter was generated from the plasmid pEBNA-G-LMP2A and cloned into the PmlI site of the plasmid pST76K-SR as described above for SFFV-hNGFR. The resulting plasmid was subsequently used for the two-step BAC mutagenesis procedure to generate MHV-68 LMP2A.

The two-step BAC mutagenesis procedure and subsequent virus reconstitution of MHV-68 hNGFR and MHV-68 LMP2A were performed as previously described [23]. All restriction enzymes except for EcoRI (Fermentas, Vilnius, Lithuania) were purchased from New England Biolabs (Ipswich, MA, USA). 

### 2.3. MHV-68 Infection and NP-CGG Immunization

For intranasal infection, 5 × 10^4^ PFU of the virus was applied. Prior to infection, the mice were anesthetized using medetomidine-midazolam-fentanyl. Intraperitoneal infection was administered using 5 × 10^5^ PFU of the virus. For immunization, 100 µg 4-hydroxy-3-nitrophenylacetyl (NP)-chicken-gamma-globulin (CGG) (Biosearch Technologies, Hoddesdon, UK) was injected intraperitoneally. The mice were analyzed 14 days post-treatment. 

### 2.4. Organ Preparation and the Determination of Cell Numbers

The lung, the spleen, two inguinal lymph nodes and in some cases mediastinal lymph nodes were prepared from each mouse. The lung was flushed with 8 mL 0.02% Heparin-Natrium in PBS during preparation to remove the blood. Organs were stored in RPMI-1640 (Gibco, Grand Island, NY, USA) supplemented with 1% fetal calf serum (Bio&Sell, Nürnberg, Germany), 2 mM L-glutamine (Gibco, Grand Island, NY, USA), 1× non-essential amino acids (Gibco, Grand Island, NY, USA), 1 mM sodium pyruvate (Gibco, Grand Island, NY, USA), 50 µM β-mercaptoethanol (Gibco, Grand Island, NY, USA), 100 U/mL penicillin and 100 g/mL streptomycin (Gibco, Grand Island, NY, USA). Total numbers of splenic cells were determined using a Neubauer cell counting chamber. Cell numbers of B cell subpopulations were recalculated using the percentages of FACS-positive cells and the total cell numbers.

### 2.5. Flow Cytometry 

Single-cell suspensions from spleen and the lymph nodes were prepared. Lung tissue was cut into slices and enzymatically digested using 0.6 mg/mL collagenase 1a (Sigma-Aldrich, St. Louis, MO, USA) and 0.43 KU/mL DNAse I (Sigma-Aldrich, St. Louis, MO, USA) for 30 min at 37 °C before preparation of the single-cell suspension. Erythrocytes of the splenic samples and the lung tissue were lysed using 1× RBC Lysis buffer (Invitrogen, Waltham, MA, USA). For the staining of proteins on the cell surface, 0.5–1.5 × 10^6^ cells were stained for 20 min in MACS buffer (Miltenyi Biotech, Bergisch Gladbach, Germany). Stained cells were usually fixed with 2% PFA in PBS (Carl Roth, Karlsruhe, Germany) prior to measurement. For intracellular stainings, the cells were fixed using 2% PFA in PBS for 10 min followed by permeabilization with 100% ice-cold methanol for 10 min. The intracellular staining was performed for 1 h at room temperature. The following fluorescence-labelled antibodies were used: CD45R/B220-PerCP (RA3-6B2), CD45R/B220-APC (RA3-6B2), CD138-PE (281-2), CD19-APC (1D3), CD19-BV510 (1D3), CD19-PE (1D3), CD21/CD35-BV421 (7G6), CD23-PE (B3B4), CD43-Biotin (S7), CD95-BV421 (JO2), GL7-PE (GL7), human CD2 (hCD2)-BV421 (RPA-2.10), human CD2 (hCD2)-FITC (REA972), IgD-APC (11-26c.2a), IgG1-PE (A85-1), IgG3-BV421 (R40-82), IgM-APC (II/41), IgM-Horizon V450 (R6-60.2), and Streptavidin (SA)-PerCP, all purchased from BD Biosciences (San Jose, CA, USA). The CAR-FITC (E1-1) antibody was purchased from Santa Cruz Biotechnology (Dallas, TX, USA). The CD19-PeVio770 (REA749), CD38-PeVio770 and human CD2 (hCD2)-PeVio770 (LT2) antibodies were all purchased from Miltenyi Biotech (Bergisch Gladbach, Germany). The human CD2 (hCD2)-APC (RPA-2.10), IgD-PE (11-26) and IgG2b-PE (polyclonal) antibodies were all purchased from Invitrogen (Carlsbad, CA, USA) and the human CD271 (hNGFR)-APC (NGFR5) was purchased from Exbio antibodies (Vestec, Czech Republic). The LMP2A FITC (14B6) antibody was provided by the Helmholtz Center Munich Core Facility Monoclonal Antibodies (Munich, Germany). Whenever possible, TOPRO-3 (Molecular Probes, Eugene, OR, USA) or a live/dead fixable blue dead cell stain kit (Invitrogen, Waltham, MA, USA) was used to exclude dead cells. The measurement was performed using LSRFortessa (BD Biosciences, San Jose, CA, USA). Results were analyzed using BD FACS Diva v8.0.1 (BD Biosciences, San Jose, CA, USA) and FlowJo Software Version 10 (FlowJo LLC, Ashland, OR, USA). Whenever possible, it was pre-gated on single live cells. 

### 2.6. The Detection of Virus-Positive Cells Using the PrimeFlow RNA Assay

A total of 2.5-5 million cells per well were stained with a live/dead fixable blue dead cell stain kit and fluorescence-labelled surface antibodies, followed by PrimeFlow RNA assay using the respective kit (Invitrogen, Waltham, MA, USA). Probes against the eight MHV-68 tRNA-like sequences [29] were designed with the help of ThermoFisher Scientific (Waltham, MA, USA). The oligos were labelled with Alexa Fluor 647. 

### 2.7. The Measurement of Viral Genomic Load by Using Quantitative Real-Time PCR 

DNA from splenic cells was isolated with the QIAmp DNA Mini Kit (Qiagen, Hilden, Germany). The quantitative real-time PCR was performed using the ABI 7300 Real Time PCR System (Applied Biosystems, Foster City, CA, USA) as described previously [32]. Oligonucleotides are shown in Supplementary Table S1.

### 2.8. Statistics

Statistical analyses were performed using GraphPad Prism 9–10 (San Diego, CA, USA). The statistical tests that were applied are indicated in the figure legends. In case of Log-normal distribution, data were log-transformed before analysis. 

## 3. Results 

### 3.1. MHV-68 hNGFR Infection of CAR//Cγ1-cre Reporter Mice Results in Cre-Mediated Recombination in B Cells

To study whether MHV-68 infection induces Cre-mediated recombination in Cγ1-cre mice, we infected the conditional transgenic reporter mouse strain CAR//Cγ1-cre, in which the human coxsackie/adenovirus receptor (CAR) was expressed as reporter (Cre-reporter) upon Cre-mediated recombination [27,31] with the recombinant MHV-68 hNGFR reporter virus (MHV-68 hNGFR). This virus contained a SFFV promotor-driven *hNGFR* gene inserted through a two-step BAC mutagenesis procedure between ORF27 and ORF29 (Supplementary Figure S1A). To confirm the hNGFR expression, NIH3T3 fibroblasts were infected with a BAC-derived wt MHV-68 (MHV-68*) or MHV-68 hNGFR. HNGFR-positive fibroblasts could be detected after infection with MHV-68 hNGFR but not with MHV-68* (Supplementary Figure S1B). Despite the absence of detectable hNGFR expression in virally infected splenic cells in vivo, we decided to use this reporter virus as a universal control for other recombinant MHV-68 strains, as they are often attenuated compared to wt MHV-68 [33].

**Figure 1:**
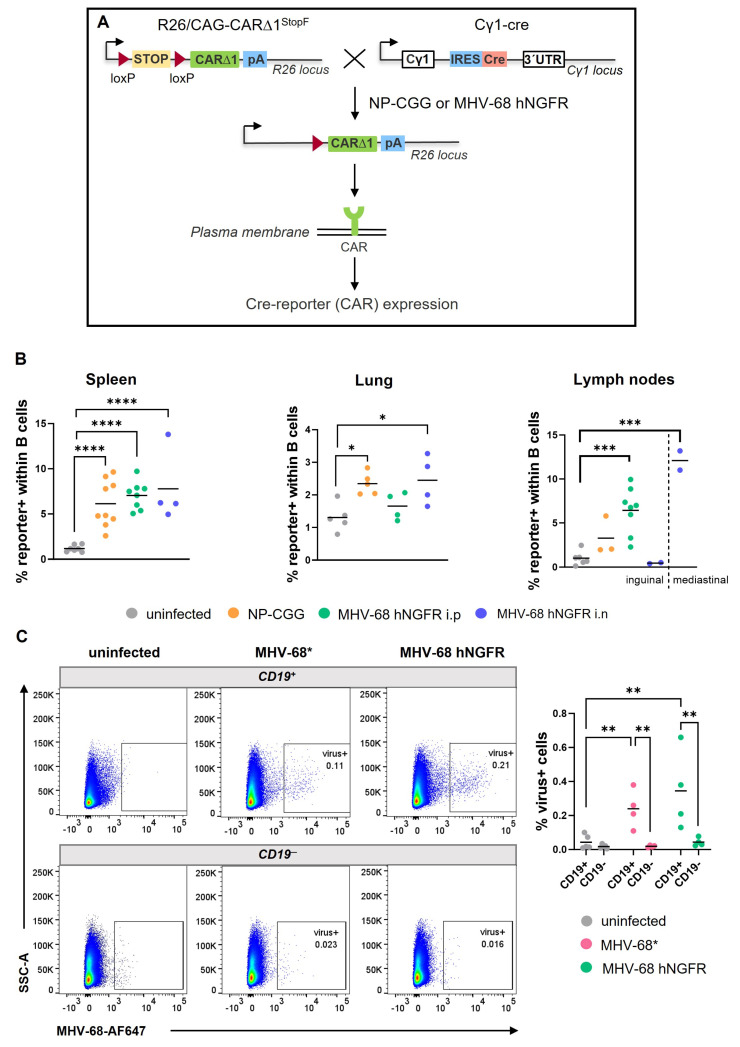
MHV-68 infection of CAR//Cγ1-cre mice leads to Cre-mediated recombination. (**A**) The breeding scheme illustrates the generation of CAR//Cγ1-cre mice. TD immunization results in Cre-recombinase expression, leading to the excision of the loxP-flanked stop cassette, and subsequent CAR-reporter expression (Cre-reporter). (**B**) The graphs depict the percentages of Cre-reporter+ B cells in the spleen, lung and lymph nodes of uninfected mice (grey), those administered NP-CGG (orange), and those infected with MHV-68 via i.p. (green) or i.n. (blue) route. Values of inguinal (left) and mediastinal (right) lymph nodes, analyzed after i.n. infection, are separated by a dotted line. Lymphocytes were pre-gated on CD19^+^ B cells (see gating strategy in Supplementary Figure S2). (**C**) In the FACS plots, the gating strategy of MHV-68+ cells within CD19^+^ or CD19^−^ cells is shown, assessed by the PrimeFLow RNA Assay 14 days post-infection of CAR//Cγ1-cre mice with MHV68* (pink) and MHV-68 hNGFR (green). Uninfected splenic cells (grey) from CAR//Cγ1-cre mice served as controls. The graph compiles the percentages of virus+ cells within CD19^+^ or CD19^−^ splenic cells from independent experiments. The horizontal lines indicate the respective means. Each symbol represents one individual mouse with *n* = 7 (uninfected), *n* = 9 (NP-CGG), *n* = 8 (MHV-68 i.p.), *n* = 4 (MHV-68 i.n.) [Spleen], *n* = 5 (uninfected), *n* = 5 (NP-CGG), *n* = 4 (MHV-68 i.p.), *n* = 4 (MHV-68 i.n.) [Lung], *n* = 6 (uninfected), *n* = 3 (NP-CGG), *n* = 8 (MHV-68 i.p.), *n* = 2 (MHV-68 i.n. inguinal), *n* = 2 (MHV-68 i.n. mediastinal) [Lymph nodes] in (**B**), and *n* = 5 (uninfected) and *n* = 4 (MHV-68* or MHV-68 hNGFR) in (**C**). The one-way (**B**) or two-way ANOVA (C) with Tukey’s multiple comparisons test was used for statistics; * *p* < 0.05, ** *p* < 0.01, *** *p* < 0.001, **** *p* < 0.0001. MHV-68^*^ = BAC-derived wildtype MHV-68.

In Cγ1-cre mice, where the Cre-recombinase was positioned downstream of the IgHCγ1-promoter, Cre expression could be induced by TD-immunization (e.g., NP-CGG) resulting in the recombination of loxP-flanked regions predominately in GC B cells. Notably, Cre expression was not limited to IgG1-switched cells but occurred in all GC B cells [27]. In CAR//Cγ1-cre mice, Cre-mediated recombination led to the removal of the stop cassette upstream of the *car*-reporter gene resulting in CAR expression on the cell surface, detectable by flow cytometry (Figure 1A). To investigate whether MHV-68 infection induces loxP-mediated recombination akin to NP-CGG immunization, we infected and immunized CAR//Cγ1-cre mice in parallel. Both the antigen and the virus were administered intraperitoneally (i.p.) to directly compare NP-CGG immunization and MHV-68 hNGFR infection. Additionally, an intranasal (i.n.) infection was included to explore whether the route of infection influenced loxP-mediated recombination.

Fourteen days after infection or immunization, the percentage of CAR-expressing cells (Cre-reporter+) was analyzed in various organs. In the spleen, the frequency of Cre-reporter+ B cells increased after MHV-68 infection, regardless of the infection route, like that after NP-CGG immunization, reaching up to 6–8% reporter+ cells. In the lung tissue, MHV-68 i.n. infection and NP-CGG immunization led to significantly higher percentages (around 2.4%) of Cre-reporter+ cells in comparison to untreated mice (~1.6%), while MHV-68 i.p infection did not lead to an increase in Cre-reporter+ cells (Figure 1B). In draining lymph nodes, the percentage of Cre-reporter+ B cells was clearly dependent on the infection route. For instance, inguinal lymph nodes exhibited an increase in Cre-reporter+ B cells only after i.p. infection (~6%) but not after i.n. infection. In contrast, i.n. infection resulted in a marked rise in the frequency of Cre-reporter+ B cells in the mediastinal LN (~12%). In summary, our findings demonstrate that MHV-68 infection in CAR//Cγ1-cre mice triggers Cre-mediated recombination in B cells of the spleen, and depending on the infection route, in the lung and some lymph nodes. 

To identify virus-infected cells, we adapted the PrimeFlow RNA Assay [28] for MHV-68 by using probes directed against the eight small t-RNA-like sequences of MHV-68 [29]. As these t-RNA-like sequences are expressed during both lytic replication and latency, the detection of MHV-68 using this method should be independent of the infection status. We successfully detected MHV-68-infected B cells (CD19^+^) while the virus was not observed in non-B cells. The percentage of virus+ CD19^+^ cells was approximately 0.3% and was comparable between MHV-68*- and MHV-68 hNGFR-infected mice (Figure 1C). 

### 3.2. MHV-68 hNGFR Infection Results in Cre-Reporter Expression in GC B Cells and Isotype-Switched Cells 

**Figure 2:**
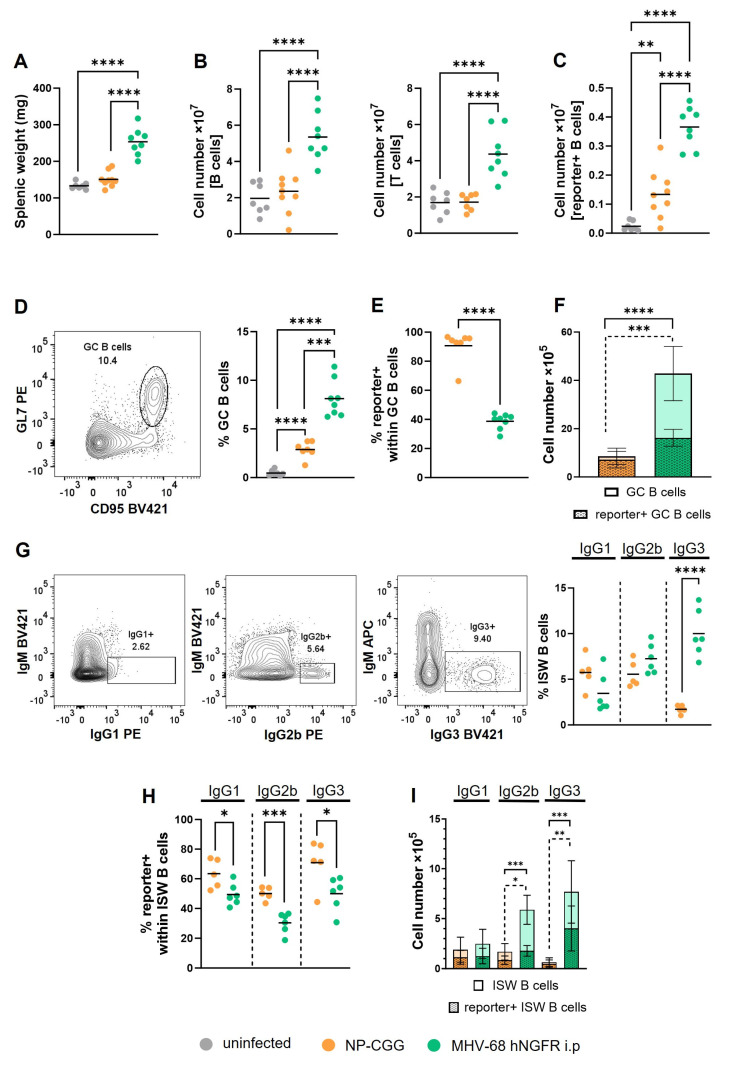
MHV-68 infection results in Cre-reporter+ GC B cells as well as in Cre-reporter+ isotype-switched B cells. (**A**) Splenic weight in mg. (**B**) The graph shows the total cell numbers of CD19^+^ B cells (left panel) and of T cells (right panel) in the spleen. (**C**) The total cell number of Cre-reporter+ B cells is displayed. The gating strategy for (Cre-reporter+) B and T cells is shown in Supplementary Figure S2. (**D**) An exemplary gating strategy for GL7^high^CD95^high^ GC B cells after MHV-68 hNGFR infection is shown in the FACS plot (left) and the percentages of total GL7^high^CD95^high^ GC B cells are displayed in the graph (right). (**E**) The percentages of Cre-reporter+ cells within GC B cells are shown. (**F**) The graph depicts the cell numbers of total GC B cells and Cre-reporter+ within total GC B cells. GC B cells were pre-gated on B220^+^ B cells. A more detailed exemplary gating strategy for (**D**–**F**) is shown in Supplementary Figure S3A. (**G**) Exemplary gating plots of IgG1-(left), IgG2b-(middle), or IgG3-(right) isotype switched (ISW) B cells after MHV-68 hNGFR infection are shown. The graph depicts the percentages of total IgG1^+^, IgG2b^+^ or IgG3^+^ isotype-switched B cells. (**H**) Percentages of Cre-reporter+ cells within IgG1^+^, IgG2b^+^ or IgG3^+^ isotype-switched B cells are shown. (**I**) Cell numbers of Cre-reporter+ isotype-switched B cells within the total numbers of isotype-switched B cells are depicted. Isotype-switched cells were pre-gated on IgD^−^B220^+^IgM^−^ cells. A more detailed exemplary gating strategy is shown in Supplementary Figure S3B–D. The mean is shown in all graphs by a horizontal line or a bar. Each symbol represents one individual mouse with *n* = 7 (uninfected), *n* = 9 (NP-CGG) and *n* = 8 (MHV-68) in (**A**,**B**, left, and **C**), *n* = 7 (uninfected), *n* = 7 (NP-CGG) and *n* = 8 (MHV-68) in (**B**, right), *n* = 7 (uninfected), *n* = 7 (NP-CGG) and *n* = 8 (MHV-68) in (**D**–**F**) and *n* = 5 (NP-CGG) and *n* = 6 (MHV-68) in (**G**–**I**). One-way ANOVA with Tukey´s multiple comparisons test was used for statistics in (**A**–**D**) and the unpaired *t*-test was performed in (**E**,**F**) and for each isotype in (**G**–**I**); * *p* < 0.05, ** *p* < 0.01, *** *p* < 0.001, **** *p* < 0.0001.

In the following more detailed analysis, we focused on analyzing the spleen. Unlike NP-CGG immunization, MHV-68 hNGFR infection led to an increase in the splenic weight (Figure 2A), which was attributed to an increase in total B and T cell numbers after viral infection (Figure 2B). This increase in lymphocytes was not observed after immunization. Both immunization and infection resulted in an increase in Cre-reporter+ splenic B cells. This increase was more pronounced after viral infection than after immunization (Figure 2C). This finding indicates that like NP-CGG immunization, MHV-68 infection induces Cre-expression and results in the efficient removal of the stop cassette. In contrast to immunization, MHV-68 infection results in a higher number of Cre-reporter+ B cells. 

MHV-68 passages the GC reaction to establish latency in memory B cells approximately 14–17 days post-infection [6]. Given our primary interest in the recombination of loxP-flanked regions in GC B cells, we compared the percentages of Cre-reporter+ GC B and post-GC B cells (isotype-switched cells) in the spleen 14 days after infection or immunization.

As expected, both immunization with NP-CGG and infection with MHV-68 hNGFR led to an increase in GC B cells (3% and 8%, respectively) (Figure 2D). Strikingly, however, the percentages of Cre-reporter+ cells within GC B cells were significantly lower in MHV-68 hNGFR-infected (40%) than in NP-CGG-immunized mice (90%) (Figure 2E). Nevertheless, the total number of Cre-reporter+ GC B cells was still significantly increased after infection compared to NP-CGG immunization (Figure 2F). Thus, MHV-68 can induce Cre-mediated recombination in GC B cells and even results in a higher number of Cre-reporter+ GC B cells than NP-CGG immunization, although in contrast to NP-CGG immunization, a smaller proportion of GC B cells underwent the removal of the stop cassette. These results imply the feasibility of manipulating genes flanked by loxP sequences within the context of a γ-herpesviral infection in GC B cells.

MHV-68 establishes long-term latency in IgD^−^ B cells [21], a fraction predominately composed of memory B cells, primarily characterized by class-switching. Overall, mice subjected to NP-CGG immunization and those infected with MHV-68 hNGFR exhibited comparable percentages of IgG1-switched and IgG2b-switched B cells. However, MHV-68-infected mice displayed significantly higher percentages of IgG3-switched B cells (9%) compared to NP-CGG treated mice (2%) (Figure 2G). Like in GC B cells, the percentages of Cre-reporter+ cells within class-switched cells were significantly reduced for all analyzed isotypes in MHV-68 hNGFR-infected compared to NP-CGG-immunized mice (Figure 2H). The calculation of the total numbers of switched B cells revealed comparable numbers of Cre-reporter+ IgG1^+^ B cells but higher numbers of IgG2b- and IgG3-switched B cells after MHV-68 infection in comparison to NP-CGG immunization. (Figure 2I). These findings demonstrate that the percentages of Cre-reporter+ memory B cells (post-GC cells) are in a similar range like in GC B cells. The bias of the switched cells towards IgG2b and IgG3 in infected, in comparison to immunized, mice resulted in comparable numbers of IgG1-switched cells in immunized and infected mice but increased numbers of IgG2b- and IgG3-switched cells. These results demonstrate that virally induced Cre-reporter+ cells can be also found in memory B cells. 

**Figure 3:**
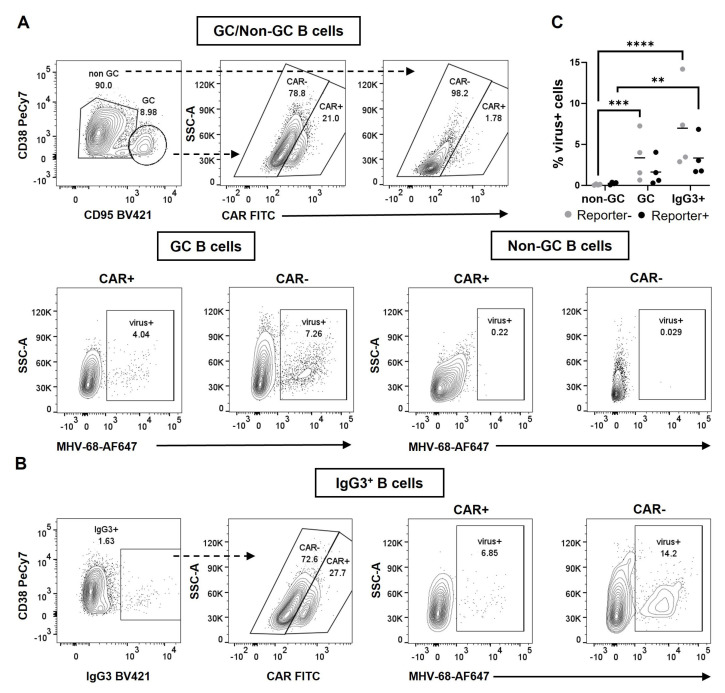
MHV-68 resides in both Cre-reporter+ and Cre-reporter− GC and IgG3-switched B cells. (**A**) Illustrations of the gating strategies used to identify virus+ cells within Cre-reporter+ or Cre-reporter− non-GC B cells or GC B cells, using CD95 and CD38 as markers, are shown. CD19 was used as B cell marker. (**B**) The gating approach for virus+ cells within Cre-reporter+ or Cre-reporter− IgG3^+^ B cells is outlined. The FACS plots depict a representative analysis of a CAR//Cγ1-cre mouse infected with MHV-68 hNGFR i.p. and analyzed 14 days post-infection. B cells were pre-gated on CD19^+^ splenic B cells. (**C**) The graph compiles the percentages of virus+ cells among Cre-reporter+ (black) or Cre-reporter− cells (grey) in the specified B cell populations. Data were determined by PrimeFlow RNA Assay followed by flow cytometry. The means are indicated by a horizontal line. Each symbol represents one individual mouse with *n* = 4 per group, and the two-way ANOVA with Tukey´s multiple comparisons test was used for statistics; ** *p* < 0.01, *** *p* < 0.001, **** *p* < 0.0001.

The two predominant B cell populations significantly expanded in MHV-68-infected in comparison to NP-CGG-immunized mice were GC B cells and IgG3-switched cells. To explore the potential accumulation of MHV-68 within these B cell subsets, we determined the percentages of virus+ cells using the PrimeFlow RNA Assay. The results revealed higher percentages of virus+ B cells in GC and IgG3-switched B cells than in non-GC B cells, indicating viral enrichment in the cell types crucial for the establishment (GC B cells) and maintenance (IgG3-switched cells) of latency. In all examined populations, the percentages of virus+ cells were evenly distributed between Cre-reporter+ and Cre-reporter− B cells (Figure 3A–C). These findings suggest that the virus may not indispensably require cell entry to trigger the removal of the stop cassette, or it might be lost in rapidly proliferating GC B cells. Moreover, these results underscore that MHV-68 entry into a cell does not necessarily ensure Cre-mediated recombination.

### 3.3. The Insertion of an EBV Gene into MHV-68 to Investigate Its Effect in the Context of a Natural Infection

**Figure 4:**
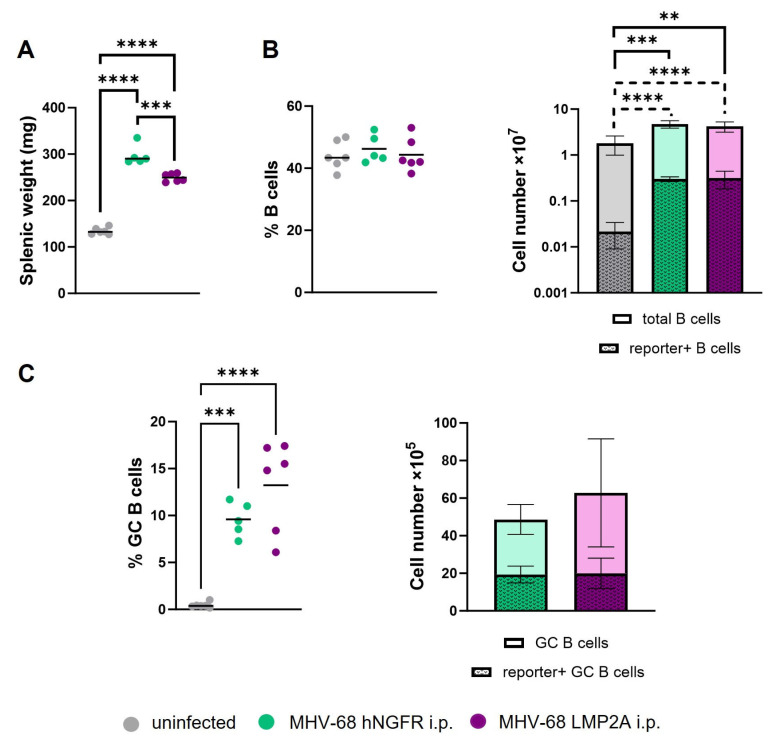
MHV-68 LMP2A infection leads to similar numbers of B and GC B cells compared to MHV-68 hNGFR infection. CAR//Cγ1-cre mice were intraperitoneally infected with either MHV-68 LMP2A (violet) or MHV-68 hNGFR (green) and splenic cells were analyzed 14 days post-infection. (**A**) The graph displays the splenic weight in mg. (**B**) The percentages of CD19^+^ B cells are shown in the left graph. The stacked graph illustrates the cell numbers of total B cells and Cre-reporter+ within total B cells. (**C**) The percentages of GC B cells, gated as B220^+^GL7^high^CD95^high^, are displayed in the left graph. The stacked graph presents numbers of total and Cre-reporter+ GC B cells. GC B cells were gated as shown in Figure 2D and Supplementary Figure S3A. The horizontal line or the bar indicates the mean. Each symbol represents one individual mouse with *n* = 6 (uninfected), *n* = 5 (MHV-68 hNGFR) and *n* = 6 (MHV-68 LMP2A). The one-way ANOVA with Tukey´s multiple comparisons test was used for statistics in (**A**–**C**, left panel) and the unpaired *t*-test in (**C**, right panel); ** *p* < 0.01, *** *p* < 0.001, **** *p* < 0.0001.

To test whether our experimental system can also be used to investigate the effects of key viral genes of EBV or KSHV (which have no homology in MHV-68) in the context of natural infection, we engineered a recombinant MHV-68, expressing the EBV protein LMP2A, as a proof of concept. The coding sequence of LMP2A, controlled by the HCMV promoter, was inserted into the MHV-68 genome between ORF27 and ORF29 through a two-step BAC mutagenesis procedure (Supplementary Figure S4A). The expression of LMP2A was confirmed by a FACS analysis of NIH3T3 fibroblasts infected with MHV-68* or MHV-68-LMP2A (Supplementary Figure S4B). The infection of CAR//Cγ1-cre mice with MHV-68 LMP2A resulted in a splenomegaly 14 days after infection although to a lesser extent compared to MHV-68 hNGFR (Figure 4A). We did not detect LMP2A expression in MHV-68 LMP2A-infected B cells by FACS, likely due to the antibody sensitivity. However, the milder splenomegaly in MHV-68 LMP2A-infected mice suggests that LMP2A was expressed and influenced latency establishment. Nevertheless, comparable percentages of total B cells (Figure 4B, left panel) and similar total B cell- and Cre-reporter+ B cell numbers (Figure 4B, right panel) were observed after MHV-68 LMP2A and MHV-68 hNGFR infection, respectively. In addition, we examined the percentages of GC B cells (Figure 4C, left panel) and the numbers of total GC B- and Cre-reporter+ GC B cells (Figure 4C, right panel). Fourteen days after infection, a slight increase in the percentage and total number of GC B cells was noted after MHV-68 LMP2A infection compared to infection with MHV-68 hNGFR. However, the statistical significance was hindered by high variation in the MHV-68 LMP2A-infected group. In conclusion, our data reveal that the construction of recombinant MHV-68, expressing genes from EBV or KSHV, is feasible. However, there is a need for additional improvements in the expression levels within the viral context. This enhancement could be accomplished by employing alternative promoters or by implementing conditional expression strategies for the EBV and KSHV genes within MHV-68.

### 3.4. The Induction of Constitutively Active CD30 Signaling via MHV-68 Infection Results in the Expansion of CD43^+^CD23^low^ B Cells

**Figure 5:**
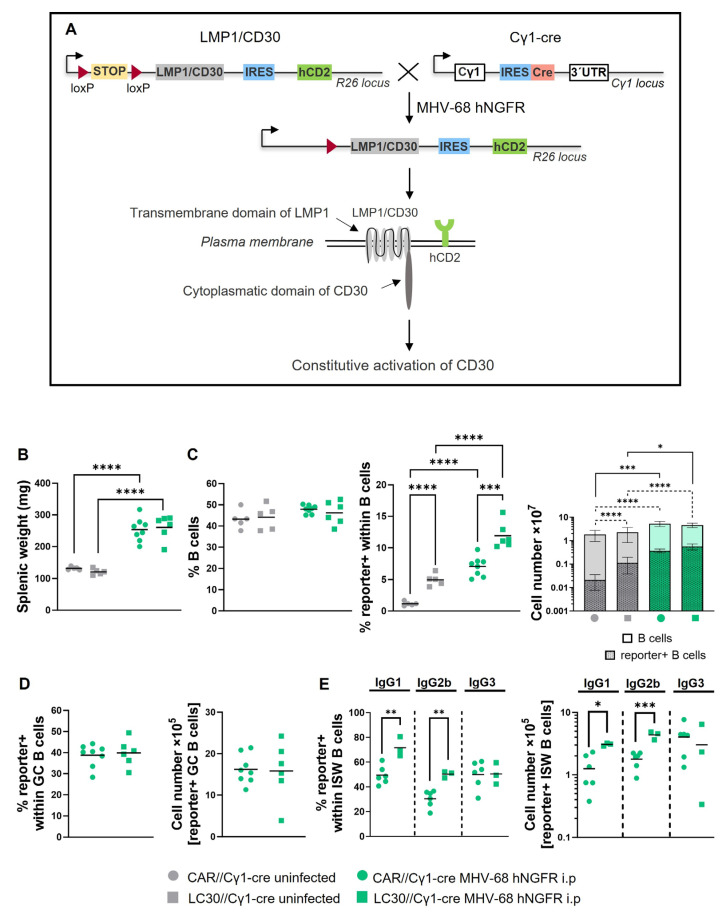
Virally induced constitutively active CD30 signaling results in more Cre-reporter+ cells within B cells. CAR//Cγ1-cre mice (dots) and LC30//Cγ1-cre mice (squares) were i.p. infected with MHV-68 hNGFR and splenic cells were analyzed 14 days post-infection. (**A**) Breeding scheme of LC30//Cγ1cre mice. The excision of the loxP-flanked stop cassette was followed by hCD2 reporter expression (Cre-reporter) and ligand-independent constitutively active CD30 signaling. (**B**) The splenic weight in mg is displayed. (**C**) The graphs summarize the percentages of B cells (left graph) and the percentages of Cre-reporter+ cells within B cells (middle graph). Cell numbers of Cre-reporter+ B cells within the total numbers of B cells are depicted in the right graph. CD19 was used as B cell marker. The gating strategy is shown in Supplementary Figure S2. (**D**) Percentages of Cre-reporter+ cells within GC B cells and total cell numbers of Cre-reporter+ GC B cells are depicted and summarized in the graphs. GC B cells were gated as B220^+^GL7^high^CD95^high^ cells as shown in Figure 2D and Supplementary Figure S3A. (**E**) Percentages and total cell numbers of Cre-reporter+ cells within IgG1^+^, IgG2b^+^ or IgG3^+^ isotype-switched (ISW) B cells are depicted. ISW cells were pre-gated on IgD^−^B220^+^IgM^−^ cells as shown in Figure 2G and Supplementary Figure S3B–D. The mean is shown in all graphs as a horizontal line or as a bar. Each symbol represents one individual mouse with *n* = 5 (uninfected, CAR//Cγ1-cre), *n* = 5 (uninfected, LC30//Cγ1-cre), *n* = 8 (MHV-68, CAR//Cγ1-cre) and *n* = 6 (MHV-68, LC30//Cγ1-cre) in (**B**–**D**) and *n* = 6 (CAR//Cγ1-cre) and *n* = 3 (LC30//Cγ1-cre) in (**E**). The two-way ANOVA with Tukey´s multiple comparisons test (**B**,**C**) or unpaired *t*-test (**D**,**E**) was used for statistics; * *p* < 0.05, ** *p* < 0.01, *** *p* < 0.001, **** *p* < 0.0001.

As an example of deregulated cellular gene expression, we chose enhanced CD30 signaling, known to be triggered by EBV infections [34,35]. To explore this, we compared LMP1/CD30//Cγ1-cre (LC30//Cγ1-cre) and CAR//Cγ1-cre mice, both infected with MHV-68 hNGFR. Cells that eliminated the stop cassette upon MHV-68 hNGFR infection in LC30//Cγ1-cre mice expressed the fusion protein LMP1/CD30, encoding for a constitutively active CD30 receptor and the Cre-reporter hCD2 (Figure 5A). 

Fourteen days post-infection, both LC30//Cγ1-cre and CAR//Cγ1-cre mice exhibited a comparable splenomegaly and B cell percentages (Figure 5B,C), while the percentage of Cre-reporter+ B cells (11%) was higher in LC30//Cγ1-cre mice (Figure 5C, middle). This elevation did not result in a significant increase in total or Cre-reporter+ B cell numbers (Figure 5C, right). Uninfected LC30//Cγ1-cre mice (5%) showed a higher proportion of Cre-reporter+ B cells than uninfected CAR//Cγ1-cre mice (1%), suggesting the spontaneous deletion and subsequent proliferation of Cre-reporter+ cells over time. These findings imply that the LMP1/CD30 expression in Cre-reporter+ B cells induces B cell proliferation, consequently elevating the percentage of reporter+ B cells (Figure 5C).

In the subsequent analysis, we sought to identify which B cell populations contributed to the heightened percentages of Cre-reporter+ cells in LC30//Cγ1-cre mice. While the percentages and total B numbers of Cre-reporter+ cells in GC B cells were similar (Figure 5D), there was an increase in isotype-switched IgG1 and IgG2b cells. This suggests that LMP1/CD30 expression in the GC B cells enhances the switching towards IgG1 and IgG2b (Figure 5E). 

**Figure 6:**
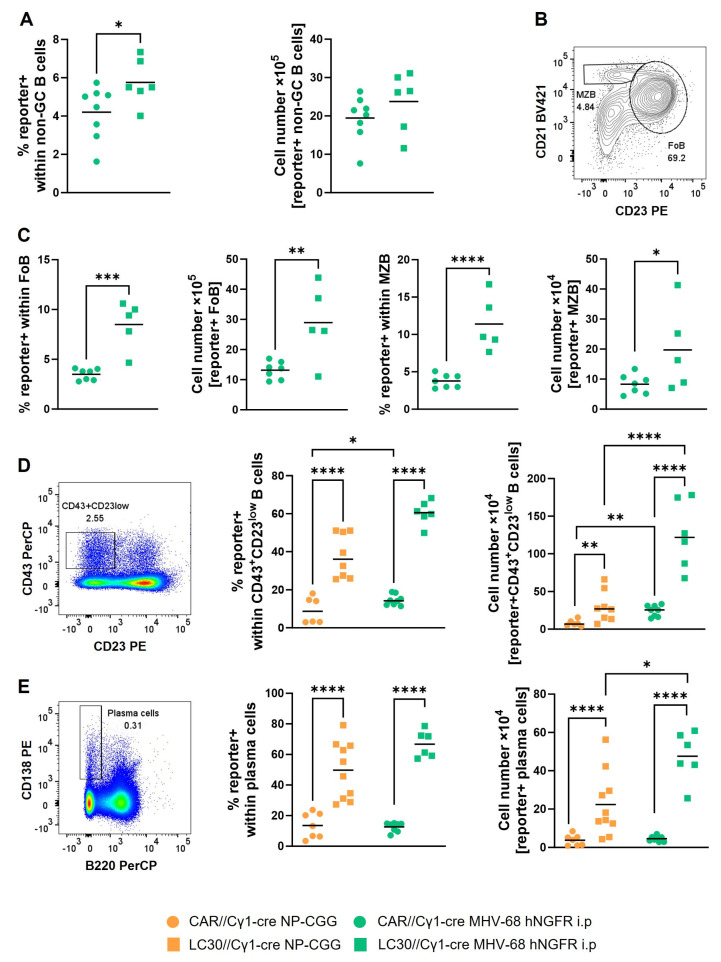
MHV-68 infection of LC30//Cγ1-Cre mice results in more Cre-reporter+ non-GC B cells. CAR//Cγ1-cre mice (dots) and LC30//Cγ1-cre mice (squares) were intraperitoneally infected with MHV-68 hNGFR and splenic cells were analyzed 14 days post-infection (green) or after NP-CGG immunization (orange). (**A**) The graphs illustrate the percentages of Cre-reporter+ cells within non-GC B cells and total cell number of Cre-reporter+ non-GC B cells. Non-GC B cells were determined as B220^+^GL7^−^CD95^low/mid^ cells with the gating strategy depicted in Supplementary Figure S3A. (**B**) The gating strategy of MZB and FoB cells, using CD23 and CD21 as markers, is presented in a representative plot of a CAR//Cγ1-cre mouse intraperitoneally infected with MHV-68 hNGFR. The cells were pre-gated on CD19^+^ B cells. A more detailed gating strategy for Cre-reporter+ MZB and FoB cells can be found in Supplementary Figure S5A. (**C**) Percentages and cell numbers of Cre-reporter+ MZB and FoB cells are shown, with FoB cells on the left and MZB cells on the right. (**D**) On the left side, an exemplary gating strategy for CD43^+^CD23^low^ B cells from a MHV-68 hNGFR-infected CAR//Cγ1-cre mouse is shown. The cells were pre-gated on CD19^+^ B cells. A more detailed gating strategy can be found in Supplementary Figure S5B. The middle graph illustrates the percentages of Cre-reporter+ cells within CD43^+^CD23^low^ B cells and the right graph shows total cell numbers of Cre-reporter+ CD43^+^CD23^low^ B cells. (**E**) Plasma cells (PC) were identified as CD138^+^B220^−^ lymphocytes. The left panel presents an exemplary gating strategy with cells from a MHV-68 hNGFR intraperitoneally infected CAR//Cγ1-cre mouse, with detailed information about the gating strategy available in Supplementary Figure S5C. The middle graph displays the percentages of Cre-reporter+ cells within PC, while the right graph shows total cell numbers of Cre-reporter+ PC. The mean is shown in all graphs by a horizontal line. Each symbol represents one individual mouse with *n* = 8 (CAR//Cγ1-cre) and *n* = 6 (LC30//Cγ1-cre) in (**A**), *n* = 7 (CAR//Cγ1-cre) and *n* = 5 (LC30//Cγ1-cre) in (**C**), *n* = 6 (NP-CGG, CAR//Cγ1-cre), *n* = 8 (NP-CGG, LC30//Cγ1-cre), *n* = 8 (MHV-68, CAR//Cγ1-cre) and *n* = 6 (MHV-68, LC30//Cγ1-cre) in (**D**) and *n* = 7 (NP-CGG, CAR//Cγ1-cre), *n* = 10 (NP-CGG, LC30//Cγ1-cre), *n* = 8 (MHV-68, CAR//Cγ1-cre) and *n* = 6 (MHV-68, LC30//Cγ1-cre) in (**E**). An unpaired *t*-test (**A**–**C**) or two-way ANOVA with Tukey´s multiple comparisons test (**D**,**E**) was used to test for statistics; * *p* < 0.05, ** *p* < 0.01, *** *p* < 0.001, **** *p <* 0.0001.

The same percentages of Cre-reporter+ cells in GC prompted a closer examination of the non-GC B cell population. Subsequent analyses revealed that the heightened percentages and cell numbers of Cre-reporter+ cells in MHV-68-infected LC30//Cγ1-cre mice, compared to the control cohort, were found in the non-GC fraction, including FoB and MZB cells (Figure 6A–C). We conclude that these B2 cells were activated by T cells triggered by the viral infection. These T cells, in turn, secrete cytokines and may induce, if they come into the neighborhood of B cells, the activation of the Cγ1-promoter and the subsequent expression of Cre. These Cre-reporter+ B cells may bypass entry into the GC reaction. The highest percentages of Cre-reporter+ cells and the greatest differences between infected LC30//Cγ1-cre and CAR//Cγ1-cre mice were observed in the CD43^+^CD23^low^ B cell population (Figure 6D). Our previous work demonstrated that constitutively active CD30 signaling in B cells leads to the expansion of a CD43^+^CD23^low^ B cell population. We could demonstrate that this population comprises a mixture of B1 cells and plasma cell (PC) progenitors. This population could originate from either B1 or activated B2 cells [25]. In addition, the percentages of Cre-reporter+ cells and cell numbers were also significantly increased in plasma cells (PC) from LC30//Cγ1-cre in comparison to CAR//Cγ1-cre control mice after MHV-68 infection (Figure 6E). The augmented numbers of hCD2+ PC can originate from both the expanded CD43^+^CD23^low^ B cell population and the GC reaction. Given our earlier observation that B1 cells/plasmablasts and PC were expanded upon the immunization of LC30//Cγ1-cre mice, we investigated whether viral infection results in a more robust expansion of these two populations than immunization. Indeed, we found that Cre-reporter+ CD43^+^CD23^low^ cells as well as Cre-reporter+ PC exhibited a more substantial expansion after MHV-68 infection than upon NP-CGG immunization (Figure 6D,E). These findings offer an initial indication that cooperative effects between herpesviral infections and deregulated cellular gene expression can be studied in this mouse model.

**Figure 7:**
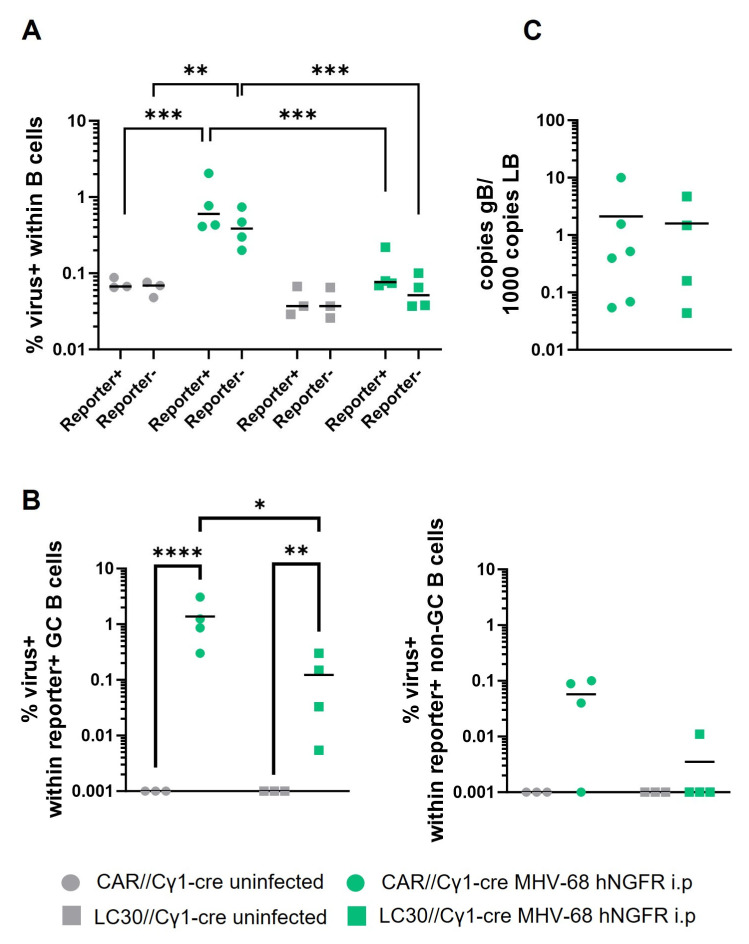
The virus+ cells are reduced in MHV-68-infected LC30//Cγ1-cre mice in comparison to CAR//Cγ1-cre mice. (**A**) Percentages of virus+ cells among Cre-reporter+ or reporter− CD19^+^ B cells. (**B**) Percentages of virus+ Cre-reporter+ GC B cells (**left**) and percentages of virus+ Cre-reporter+ non-GC B cells (**right**) are displayed. Values at 0 were set to 0.001 (instead of 0) before testing for significance. LC30//Cγ1-cre mice were compared to CAR//Cγ1-cre mice. (**C**) The viral genomic load of total splenic DNA was measured using qPCR 14 days post-infection. The mean is shown in all graphs by a horizontal line. Each symbol represents one individual mouse with *n* = 3 (uninfected, CAR//Cγ1-cre), *n* = 4 (MHV-68, CAR//Cγ1-cre), *n* = 3 (uninfected, LC30//Cγ1-cre) and *n* = 4 (MHV-68, LC30//Cγ1-cre) in (**A**), *n* = 3 (uninfected) and *n* = 4 (MHV-68) per group in (**B**) and *n* = 6 (CAR//Cγ1-cre) and *n* = 4 (LC30//Cγ1-cre) in (**C**). A two-way ANOVA (**A**,**B**) with Tukey’s multiple comparisons test or unpaired *t*-test (**C**) was used for statistics; * *p* < 0.05, ** *p* < 0.01, *** *p* < 0.001, **** *p <* 0.0001.

Determining the proportion of virus-positive cells using the PrimeFlow RNA Assay (Figure 7) led to a surprising observation: in both Cre-reporter+ and reporter− B cells, the percentages of virus-containing cells were lower in MHV-68-infected LC30//Cγ1-cre mice than those in CAR//Cγ1-cre mice, almost approaching the background levels observed in the uninfected mice (Figure 7A). Despite MHV-68 being enriched in GC B cells similar to infected CAR//Cγ1-cre mice, the percentage of virally infected cells was approximately 10-fold lower in LC30//Cγ1-cre mice (Figure 7B). Given the unexpected finding of fewer virus+ cells in LC30//Cγ1-cre mice, we conducted an additional analysis to determine the viral genomic load in the spleens of the infected mice using qPCR. Here, we observed a comparable viral genomic load in DNA isolated from the total splenocytes of both LC30//Cγ1-cre or CAR//Cγ1-cre mice (Figure 7C). The discrepancy between the two analyses could be attributed to enhanced viral replication, caused by the enhanced production of pre-plasmablasts and PC, thereby promoting viral reactivation. Viral replication leads to cell lysis, which may explain the observation of fewer virus+ cells, while the viral genomic load remains similar throughout the splenocytes.

In summary, our findings suggest that the combined impact of MHV-68 infection and CD30 signaling enhances the generation of CD43^+^CD23^low^ B cells and plasma cells compared to immunization. However, these expanded populations do not necessarily sustain MHV-68 persistence. Accordingly, the increased generation of these populations may be mediated by an indirect effect of the MHV-68 infection rather than by a supportive function of the virus in the cell. Nevertheless, our previous data revealed that constitutively active CD30 signaling (LMP1/CD30 expression) in B cells favors the development of B cell lymphomas with a CD43^+^CD23^low^ B cell phenotype [25]. It will therefore be interesting to investigate in the future whether the increased expansion of this population in young infected LC30//Cγ1-cre mice enhances lymphoma development in aged mice.

## 4. Discussion 

γ-herpesvirus infections are associated with human B cell lymphomas. To enhance our comprehension of the interplay between the cellular and viral genes in lymphoma development, we aimed to establish an animal model allowing for genetic modifications of both the herpesvirus and cellular genes. For this purpose, we selected MHV-68, a murine γ-herpesvirus, which, like EBV, establishes latency in memory B cells through passage through the GC reaction and possesses the advantage of being genetically modifiable [21,36]. Since the majority of EBV-associated B cell lymphomas arise from germinal center B cells [5], we intended to use an MHV-68 infection to activate or inactivate cellular genes through Cre-mediated recombination in GC B cells. This should be achieved by infecting conditional mice in combination with a GC-specific Cre line (Cγ1-Cre mice) with MHV-68. Here, we present insights into the feasibility and potential of this mouse model for investigating the mechanisms underlying EBV-induced lymphoma development.

Our initial investigation focused on determining whether MHV-68 could induce Cre-mediated recombination in B cells from CAR//Cγ1-cre mice. After MHV-68 infection, we observed the presence of B cells expressing the Cre-reporter CAR in the lymph nodes, spleen, and the lung, indicating that MHV-68 effectively induces Cre-mediated recombination in Cγ1-Cre mice. A notable discrepancy in infection routes and the appearance of Cre-reporter+ B cells became evident when examining different types of lymph nodes. In inguinal lymph nodes (LN), Cre-reporter+ B cells were detected only after intraperitoneal, but not after intranasal, administration of the virus. Conversely, after intranasal infection, Cre-reporter+ B cells were found in the mediastinal LN. Since we assume that the appearance of Cre-reporter+ cells is linked to the presence of the virus in the corresponding organs, we attribute this distinction to varying dissemination patterns of the virus following intranasal and intraperitoneal infection. Previous studies using recombinant MHV-68 expressing a fluorescent marker or a luciferase gene provided evidence that the mode of inoculation affects which organs or cell types are initially infected and how viruses subsequently spread [37,38]. The intranasal infection of MHV-68 results in virus entry into the lung, where it replicates, before migrating to the mediastinal LN. The virus is subsequently transported to the spleen and other draining LN through infected B cells, the spleen being one of the first organs to be invaded by infected B cells. Intraperitoneal injection allows MHV-68 to bypass mucosal barriers, facilitating faster dissemination [39]. Consequently, we hypothesize that 14 days after infection, the virus will be present in inguinal LN after intraperitoneal, but not after intranasal infection. 

To enable the identification of virus-positive B cells, we modified the PrimeFlow RNA Assay [28] for the detection of t-RNA-like sequences specific to MHV-68. These RNAs are expressed independently of the lytic or latent state of the virus [29]. A comparable approach has been successfully employed to detect the EBV genome in human blood and bone marrow samples using probes targeting Epstein–Barr Virus-encoded small RNAs (EBERs), known to be expressed upon EBV infection regardless of the latency state [30,40]. The PrimeFlow RNA Assay demonstrated reliability in our investigation, revealing that 0.3% of splenic CD19^+^ B cells were MHV-68-positive 14 days post-infection (Figure 1C). This percentage of virus-positive cells aligns with previous reports where percentages of virus-positive cells (YFP^+^) were determined in mice infected with a YFP-reporter virus [36,41]. Notably, we predominantly detected the virus in CD19^+^ cells, supporting the existing evidence that MHV-68 primarily establishes latency in B cells [6,21]. In conclusion, our Cre-mediated reporter system, in combination with the PrimeFlow RNA Assay, could be a promising new tool for a more detailed exploration of the initial sites of virus emergence and its temporal spread, depending on the route of infection.

Given that the spleen is the main reservoir of MHV-68 around 14–16 days post-infection, our investigation focused on this organ. We found an increased splenomegaly and a higher percentage of Cre-reporter+ cells after infection compared to immunization. Given the expected expansion of lymphocytes after viral infection, this phenomenon could arise from either the induction of Cre-expression in a larger population of B cells or a more robust proliferation of Cre-reporter-expressing cells in the context of viral infection. Furthermore, we identified a more pronounced increase in total and Cre-reporter+ GC B cells in MHV-68-infected compared to NP-CGG-immunized CAR//Cγ1-cre mice (Figure 2F), pointing towards an intensified immune reaction following viral infection. Intriguingly, the percentages of Cre-reporter+ cells within GC B cells were lower after viral infection than after immunization, suggesting that not every cell entering the germinal center in response to the viral infection expressed the Cre-reporter. This discrepancy may be attributed to the distinct immune responses triggered by the two stimuli. Immunization with NP-CGG typically induces a Th2 immune response, characterized by IL-4 secretion and switching to IgG1 [42,43]. In contrast, MHV-68 infection elicits a Th1 immune response with interferon-γ secretion [44], leading preferentially to IgG2 and IgG3-switched B cells [36]. Consistent with this, our observation revealed that MHV-68 infection resulted in more IgG2- and IgG3-switched cells and reduced IgG1-switched cells in comparison to NP-CGG immunization. A similar observation was reported recently by another group [36]. The variance in cytokine production between infected and immunized mice may explain the diminished deletion efficiency of the stop cassette in GC B cells after viral infection. Although in Cγ1-Cre mice, the *cre*-transgene is under the control of the Cγ1-promoter, Cre expression does not only occur in IgG1-switched cells but expands on all GC B cells, resulting in Cre-reporter expression in all isotype-switched cells [27]. Nevertheless, it is conceivable that a Th2 response, targeting preferentially the CHγ1-promoter, may induce a higher Cre expression than a Th1 response. Consequently, fewer GC B cells in infected mice may reach the amount of Cre necessary for Cre-mediated recombination in comparison to immunized mice. It would be interesting to investigate whether the infection of other GC-specific Cre strains such as AID-Cre or S1pr2-Cre [45,46] results in an equal deletion efficiency after immunization and infection. 

Using the PrimeFlow technique, we detected around 2–3% virus-containing GC B cells. This observation aligns with the findings of two prior studies, where the percentage of virus-positive cells among total GC B cells ranged from 2 to 10% [36,41]. Interestingly, MHV-68 exhibited enrichment in both Cre-reporter+ and Cre-reporter− GC B cells. The existence of the virus in Cre-reporter− cells suggests that the entry of the virus does not guarantee the deletion of the stop cassette, whereas Cre-reporter+ cells that were virus− indicate that the virus does not necessarily need to enter the cell to induce the expression of Cre. Our interpretation suggests that T cell activation triggered by the viral infection and subsequent cytokine secretion is the main player in activating the CHγ1-promoter, leading to Cre expression. Notably, we also observed GC B cells that both expressed the reporter and were virus positive. These double-positive GC B cells are crucial for our study as they enable the investigation of a potential interaction between deregulated cellular gene expression and viral infection in B cell lymphomagenesis. While the number of double-positive cells in our system is relatively low, we find it well correlated to EBV. Thus, in acute EBV infection, only a limited number of EBV-infected cells (10–20 cells per GC) are detected in the GC [47]. Nevertheless, these rare numbers of EBV-infected cells in the GC increase the risk of B cell lymphoma development by acquiring mutations in cellular genes, and potentially experience the deregulation of viral gene expression. Given that our model opens the possibility to mimic mutations and/or the deregulation of cellular and viral genes in GC B cells in advance, the presence of a few double-positive cells may be sufficient to ultimately induce lymphoma development in transgenic mice.

To assess the impact of a recombinant MHV-68 in our mouse model, we inserted LMP2A, a viral gene commonly expressed in EBV^+^ lymphomas, into MHV-68. At day 14 post-infection, we did not observe clear differences in total B cell numbers in the spleens of mice infected with MHV-68 LMP2A compared to those infected with MHV-68 hNGFR. However, there was a noticeable trend towards an increased population of GC B cells in MHV-68 LMP2A-infected mice compared to MHV-68 hNGFR-infected mice (Figure 4C). While this difference did not reach statistical significance at this early stage after infection (“early latency”), future studies examining later time points during “late latency” will be of great interest. LMP2A is known for its ability to mimic the BCR, providing survival signals even in the absence of the BCR [17,19]. This capability enables LMP2A to rescue GC cells with crippled mutations [18,19]. Moreover, LMP2A expression in B cells results in the upregulation of genes associated with cell cycle induction and inhibition of apoptosis [48], and promotes spontaneous GC formation in Peyer’s patches in the absence of BCR expression [49]. It is plausible that virally infected cells undergo substantial expansion upon re-entering the GC reaction. In such a scenario, LMP2A might confer a survival advantage to GC B cells, potentially rescuing cells that would otherwise face negative selection. These cells, escaping negative selection, could have an increased risk of developing into B cell lymphomas.

Many EBV^+^ B cell lymphomas exhibit high levels of CD30 [2,3,4], prompting the hypothesis that CD30 signaling and γ-herpesviral infection cooperatively contribute to B cell lymphomagenesis. To explore this, we induced the expression of a LMP1/CD30 fusion protein, encoding a constitutively active CD30 receptor, through MHV-68 infection. Infected LC30//Cγ1-cre mice showed an increased population of Cre-reporter+ B cells compared to the controls (Figure 5C), in accordance with our previous observation that constitutive CD30 signaling exerts a proliferative effect in B cells [25]. Interestingly, the expanded population of Cre-reporter+ cells was contained in the non-GC B cell fraction, including FoB, MZB, and memory B cells, but not in the GC fraction. FoB and MZB cells might undergo activation and a subsequent expression of Cre during the acute infection of MHV-68, wherein infected B cells traverse the marginal zone to enter the B cell follicle for the dissemination of the virus in the spleen [50]. In addition, B cells that were activated either directly or indirectly (by T cells) during MHV-68 infection may partially exhibit a CD43^+^CD23^low^ phenotype. This could explain the high percentage of Cre-reporter+ cells in this population and its augmented expansion compared to that in immunized mice. 

Unexpectedly, we observed a 10-fold decrease in virus+ B cells in infected LC30//Cγ1-cre mice compared to CAR//Cγ1-cre mice. Our recent findings have demonstrated that constitutively active CD30 signaling enhances plasmablast (PB)/PC differentiation. It is suggested that γ-herpesviruses undergo reactivation during PC differentiation [51]. Since we knew from our previous study that Blimp-1 and IRF4 levels are elevated in LMP1/CD30-expressing B cells [25], which are key factors for both PC differentiation and viral reactivation [6], we assumed that the lytic phase of MHV-68 is turned on upon LMP1/CD30 expression, resulting in the lysis of MHV-68+ cells. This hypothesis is supported by the qPCR result measuring the total splenic viral genomic load, including the lytic virus, which was similar in the spleens of mutant and control mice. An alternative explanation for the diminished proportion of virus+ B cells in LC30//Cγ1-cre mice could be attributed to the potential loss of the virus from initially infected cells. This hypothesis assumes that the episomal MHV-68 genome is replicated more slowly compared to the actively proliferating LMP1/CD30-expressing cells. Our hypothesis aligns with a previous publication, where MHV-68 genomes were tracked in cells that aberrantly proliferated due to the insertion of oncogenes in vivo, resulting in the loss of the viral genome in most cells [52]. Interestingly, such a “hit-and-run mechanism” was also discussed in the context of EBV-driven B cell lymphoma [53].

In summary, we have established an animal model that enables us to study genetic modifications in GC B cells in the context of γ-herpesviral infections. A first indication of the potential of the model is the increased expansion of an activated pre-plasmablastic population in LC30//Cγ1-Cre mice after viral infection compared to immunization. Given that this population is suspected to be the precursor of lymphomas in these mice [25], it will be interesting to investigate in the future whether MHV-68 intensifies lymphoma development in this context. The examination of virus+ cells in this model could shed light on whether MHV-68 has a direct or indirect impact on lymphoma development. The presence of virus-positive lymphomas would suggest a direct contribution of MHV-68 to lymphomagenesis. Conversely, the occurrence of virus-negative lymphomas would indicate a “hit-and-run” mechanism or an indirect contribution of MHV-68 to lymphoma development, possibly through the stimulation of T cells. Employing recombinant viruses expressing LMP1 and LMP2A for the infection of transgenic LMP1/CD30 mice will enable the examination of the additional impact of crucial EBV proteins on the development of these lymphomas. We believe that our newly established mouse model holds promise as a valuable tool for studying γ-herpesviral/host interactions in the development of B cell lymphomas.

## Data Availability

The data presented in this study are available upon request from the corresponding authors.

## Supplementary Materials

The following supporting information can be downloaded at: https://www.mdpi.com/article/10.3390/cells12242780/s1, Figure S1: MHV-68 hNGFR construction and characterization; Figure S2: Gating strategy of Cre-reporter+ cells within CD19^+^ B cells or T cells is shown. A representative example of splenocytes from MHV-68 hNGFR i.p infected CAR//Cγ1-cre mice is depicted. Whenever possible, it was pre-gated on single live cells; Figure S3: Gating strategies of the percentages of Cre-reporter+ cells (A) within the B220^+^GL7^high^CD95^high^ GC B cells or B220^+^GL7^−^CD95^low/mid^ non-GC B cells or (B–D) within IgG1^+^-, IgG2b^+^-, IgG3^+^-isotype-switched splenic B cells. ISW-B cells were pre-gated on B220^+^IgD^−^IgM^−^ B cells. Representative examples of splenocytes from MHV-68 hNGFR i.p infected CAR//Cγ1-cre mice are shown (day 14 post infection); Figure S4: MHV-68 LMP2A construction and characterization; Figure S5: (A–C) Representative examples of stained splenic cells of CAR//Cγ1-cre mice 14 days after MHV-68 hNGFR i.p infection are shown. (A) Gating strategies of Cre-reporter+ cells within the CD19^+^CD21^+^CD23^−^ MZB and CD19^+^CD21^+^CD23^+^ FoB cell gate, (B) Cre-reporter+ CD43^+^CD23^low^CD19^+^ B cells and (C) Cre-reporter+ CD138^high^B220^low^ plasma cells are shown; Table S1: Oligonucleotides used for quantitative real-time PCR.
