# Supplementary material for: A Mouse Model to Study the Pathogenesis of γ-herpesviral Infections in Germinal Center B Cells
Source: Cells. 2023 Dec 6;12(24):2780. doi: 10.3390/cells12242780 (PMC10741729; doi:10.3390/cells12242780)
Supplement: Supplementary file 1 [file cells-12-02780-s001.zip › cells-2713171-supplementary.pdf]

Supplementary information

A mouse model to study the pathogenesis of γ-herpesviral infections in germinal center B cells

Ursula Rambold[1], Stefanie Sperling[2], Zakir Chew[2], Yan Wang[2], Beatrix Steer[1], Krisztina Zeller[2], Lothar J. Strobl[2,3], Ursula Zimber-Strobl[2,3*] & Heiko Adler[1,4*]

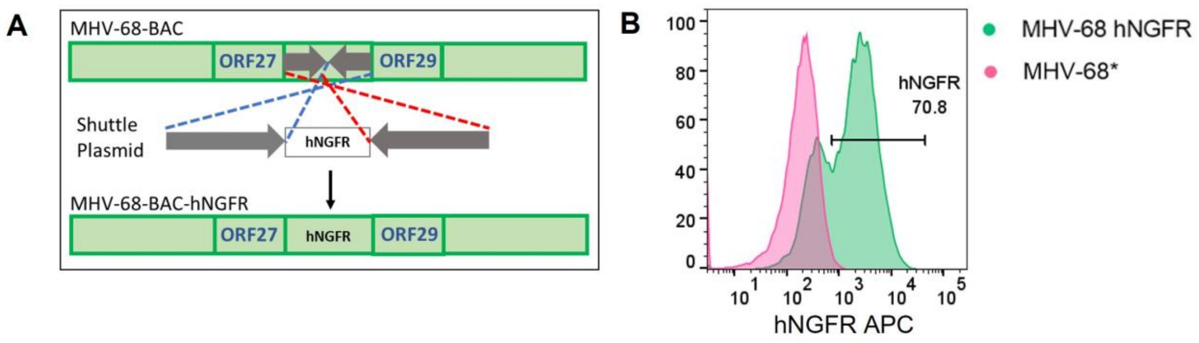

Supplementary Fig. 1: MHV-68 hNGFR construction and characterization. **(A)** Scheme of MHV-68 hNGFR construction. **(B)** NIH3T3 cells were infected with 2 M.O.I; 48 h p.i., the cells were stained with anti-hNGFR antibody and measured by flow cytometry. The histogram overlay shows the hNGFR expression in NIH3T3 cells which were either infected with MHV-68* (pink) or MHV-68 hNGFR (green) without pre-gating on other markers. The experiment was performed twice; MHV-68* = BAC-derived wildtype MHV-68.

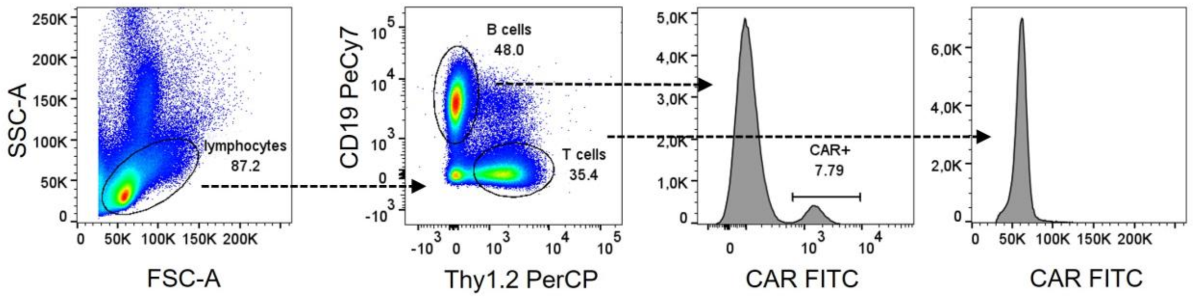

Supplementary Fig. 2: Gating strategy of Cre-reporter+ cells within CD19⁺ B cells or T cells is shown. A representative example of splenocytes from MHV-68 hNGFR i.p infected CAR//Cγ1-cre mice is depicted. Whenever possible, it was pre-gated on single live cells.

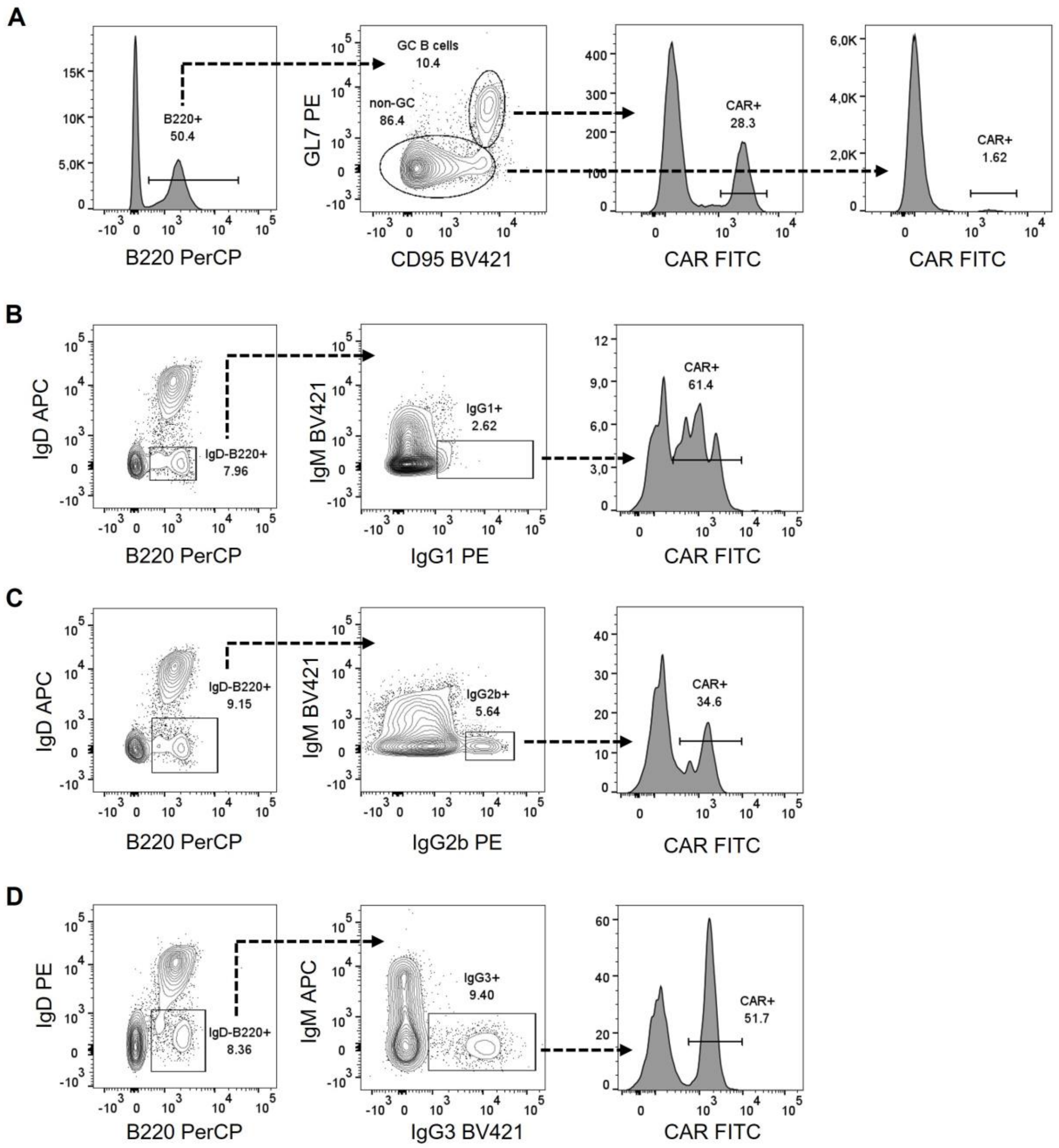

Supplementary Fig. 3: Gating strategies of the percentages of Cre-reporter+ cells **(A)** within the B220^{+}GL7^{high}CD95^{high} GC B cells or B220^{+}GL7^{-}CD95$^{low/mid}$ non-GC B cells or **(B-D)** within IgG1^{+}-, IgG2b^{+}-, and IgG3^{+}-isotype-switched splenic B cells. ISW-B cells were pre-gated on B220^{+}IgD^{-}IgM^{-} B cells. Representative examples of splenocytes from MHV-68 hNGFR i.p infected CAR//Cγ1-cre mice are shown (day 14 post infection).

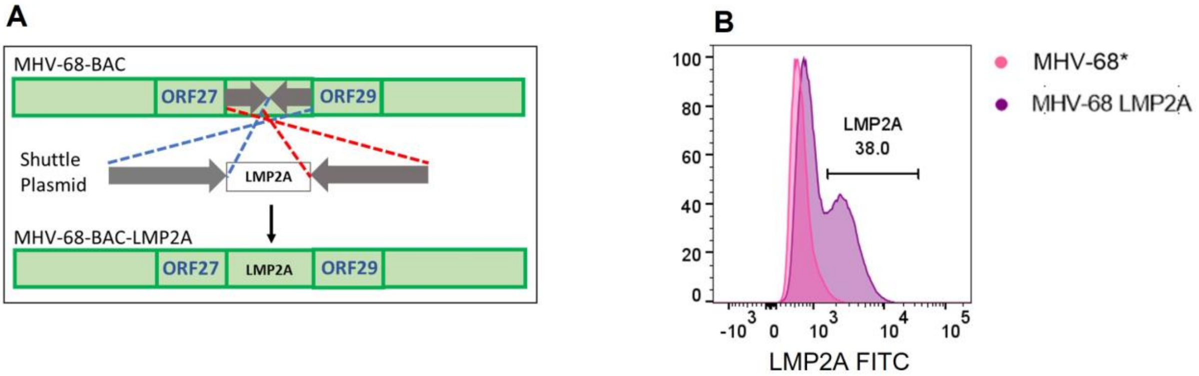

Supplementary Fig. 4: MHV-68 LMP2A construction and characterization. **(A)** Scheme of MHV-68 LMP2A construction. **(B)** NIH3T3 cells were infected with 2 M.O.I of MHV-68* or MHV-68 LMP2A. After 48 h incubation, the cells were harvested and intracellularly stained with an anti-LMP2A FITC antibody, followed by measurement by flow cytometry. The histogram overlay shows the LMP2A expression in NIH3T3 cells after infection with MHV-68* and MHV-LMP2A. MHV-68* served as control; MHV-68* = BAC-derived wildtype MHV-68.

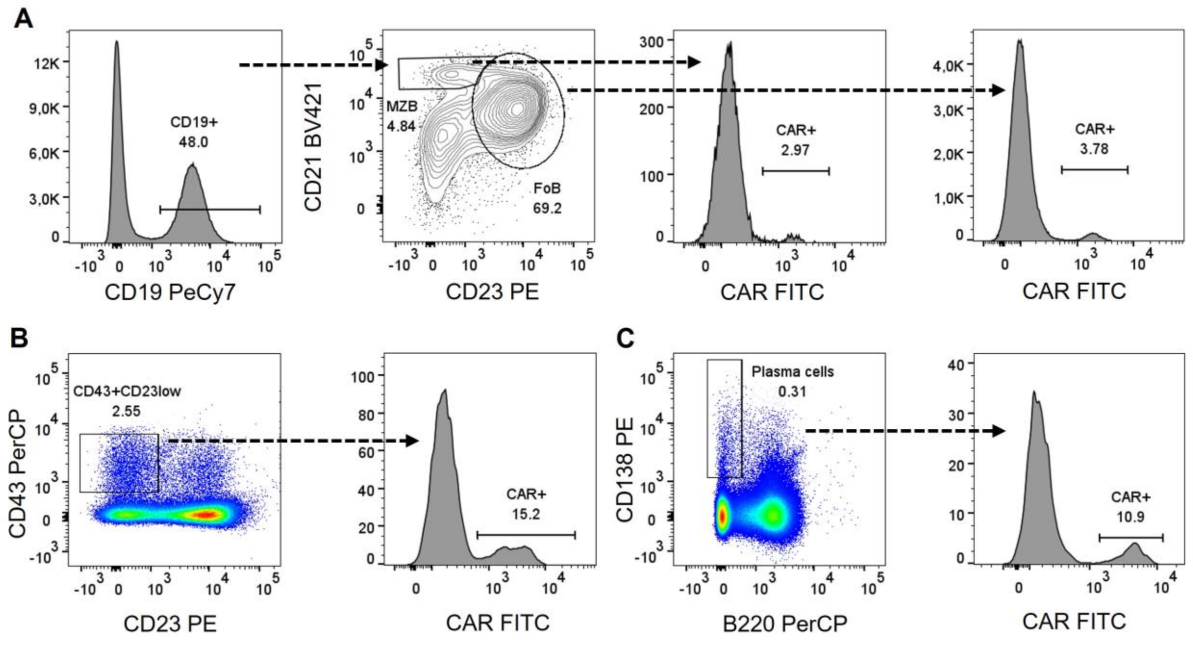

Supplementary Fig. 5: (A-C) Representative examples of stained splenic cells of CAR//Cγ1-cre mice 14 days after MHV-68 hNGFR i.p infection are shown. (**A)** Gating strategies of Cre-reporter+ cells within the $CD19^{+}CD21^{+}CD23^{-}$ MZB and $CD19^{+}CD21^{+}CD23^{+}$ FoB cell gate, **(B)** Cre-reporter+ $CD43^{+}CD23^{low}CD19^{+}$ B cells and **(C)** Cre-reporter+ $CD138^{high}B220^{low}$ plasma cells are shown.

Supplementary Table 1: Oligonucleotides used for quantitative real-time PCR

**Primer/Probe for real time PCR**	**Primer/Probe Sequence**
gB forward primer	5‘ - GGC CCA AAT TCA ATT TGC CT - 3‘
gB reverse primer	5‘ - CCC TGG ACA ACT CCT CAA GC - 3‘
gB probe	5‘ - 6-Fam-ACA AGC TGA CCA GCG TCA ACA AC-Tamra - 3‘
L8 forward primer	5‘- CAT CCC TTT GGA GGT GGT A - 3‘
L8 reverse primer	5‘- CAT CTC TTC CGA TGG TGG A - 3‘
L8 probe	5‘ - Hex-ACC ACC AGC ACA TTG GCA AAC C-BHQ-1 - 3‘
